# Movement Analysis with Inertial Measurement Unit Sensor After Surgical Treatment for Distal Radius Fractures

**DOI:** 10.1089/biores.2019.0035

**Published:** 2020-05-21

**Authors:** Benedetta Zucchi, Massimiliano Mangone, Francesco Agostini, Marco Paoloni, Luisa Petriello, Andrea Bernetti, Valter Santilli, Ciro Villani

**Affiliations:** Department of Anatomy, Histology, Forensic Medicine and Orthopedics, Sapienza University, Rome, Italy.

**Keywords:** distal radius fractures, percutaneous Kirschner wires, surface EMG, volar plate and screws, wearable medical device

## Abstract

Inertial measurement unit (IMU) has recently been used to evaluate a movement of a body segment to provide accurate information of movement's characteristics. IMU systems have been validated to successfully measure joint angle during upper limb range of motion (ROM). The study aimed to retrospectively evaluate, using an IMU, the ROM recovery of the wrist after surgical treatment for distal-radius fractures with Kirschner wire fixation (KWF) or with volar plate fixation (VPF) and screws. To assess pain in the wrist joint, muscle-fatigue (MF), and functional difficulties in activities of daily living, we evaluated the patients through patient-related wrist evaluation questionnaire (PRWE) scale, disability of the arm, shoulder and hand (DASH) scale, Hand Grip Strength (HGS), and surface electromyography (EMG). We used a single IMU composed of three-axis gyroscope, a three-axis accelerometer, and a magnetometer. We calculated the value of ROM as a percentage with respect to the unaffected wrist. We also recorded surface-EMG signals over biceps brachialis, flexor carpi radialis (FCR), extensor carpi radialis (ECR), and pronator teres muscles. Forty patients were recruited for our study. Ulnar deviation (UD) was significantly higher for VPF than for KWF (*p* = 0.017); supination was significantly higher for VPF than for KWF (*p* = 0.031). The percentage of decay of the median frequency of FCR of volar plate was significantly higher than KWF. The HGS of KWF was significantly higher than VPF. In literature, there were no significant differences between the two types of treatment at long-term follow-up. Our results demonstrate a superior efficacy of VPF in terms of ROM improvement in UD and supination, but for these patients, muscle fatigue is greater than the KWF group. Based on the data available, VPF is similar to KWF for the treatment of distal radius fractures. The IMU sensor could be used in the future to evaluate ROM after surgery during patient's rehabilitation and to compare the effects with stratified analysis regarding age and fracture type, paralleled with cost-effectiveness analysis.

## Introduction

The traditional methods used for measuring the articular range of motion (ROM) involve mechanical instruments such as goniometer and/or inclinometer.^1^ Vision-based systems can also be used. They work with optical reflective markers that are attached to the subject's limb and are thus tracked in three-dimensional (3D).^2^ Optical instruments, on the contrary, require expensive equipment, take a long time to evaluate and analyze the results and are not suitable for outpatient use in daily medical practice.^2,3^ Recently, wearable and relatively inexpensive devices, to measure ROM, have been developed and used. They are inertial measurement unit (IMU) sensors and their usefulness and effectiveness in measuring joint ROM has been demonstrated.^4^

The use of IMU is an optimal solution in terms of cost and simplicity in obtaining the measurement for kinematic assessment. IMUs composed of accelerometers, gyroscopes, and magnetometers measure rotations on the three axes expressed in degrees; therefore, if well connected to a body segment, they can measure their angular variations. It can be easily used in the outpatient setting and in daily clinical practice and allows the evaluation of a large number of parameters.^5^ The use of IMU is not limited to the evaluation of the articular ROM, but it can also evaluate the presence of compensatory movements (CM) during the execution of tests, which are movements of other anatomical districts that compensate for the movement deficit of the pathological district.^6^

Based on our knowledge, there are not many studies investigating the biomechanics of movement by comparing two different surgical techniques using IMU, and for this reason, we applied the IMU for the evaluation of articular ROM in patients who had undergone surgery for distal radius fractures, treated with closed reduction and fixation with percutaneous Kirschner wires (KWF) or open reduction and internal fixation with volar plate and screws (VPF).

The differences between the two surgical techniques have been studied extensively, but no study has presented an objective evaluation of the biomechanical differences obtained as an effect of the two different surgical approaches, which consider the articular kinematics and not static evaluation tests.^7^

After a wrist injury, resuming activities of daily living can be difficult, especially ones requiring the repetitive use of the wrist and hand. This is why we decided to evaluate another parameter that is difficult to assess clinically, such as the tendency to muscle fatigue (MF).^8^ The performance of the wrist can be examined using the surface electromyography (EMG) of the forearm muscles. The use of superficial EMG during isometric contractions allows a quantitative evaluation of an index that is connected to MF. In fact, during an isometric contraction, on the basis of the frequency analysis of the signal, it is possible to evaluate the percentage of decay of the spectrum median frequency (SMF), which is calculated in intervals (percentage change in frequency from the start to the end of the isometric contraction). During the isometric contraction, the signal frequency decreases and this decrease is related to fatigue.^9–11^

The purpose of this study was therefore to retrospectively evaluate, using an IMU applied at the wrist, the recovery after KWF and VPF surgical treatment for distal radius fractures, thereby evaluating the presence of CM. Furthermore, to assess the presence of MF, we evaluated the patients through surface-EMG. Pain in the wrist joint and functional difficulties in activities of daily living were evaluated using validated questionnaires, such the disability of the arm, shoulder, and hand (DASH), patient-related wrist evaluation questionnaire (PRWE), and The Hand Grip Strength (HGS) through a dynamometer.

## Materials and Methods

This study took place from January 2017 to September 2018 at the Department of Anatomy, Histology, Forensic Medicine, and Orthopedics—Sapienza University/University Hospital Umberto I, Rome, Italy and it was approved by the Ethics and Experimental Research Committee of Sapienza University—University Hospital Umberto I, Rome, Italy (n° 5181). Informed consent was obtained from all patients for being included in the study. For our study, we used a single IMU composed of three-axis gyroscope, a three-axis accelerometer, and a magnetometer (Fisiocomputer, Rome Italy). The IMU was fixed on the dorsal surface of the hand at level of the third metacarpal bone; we positioned the IMU in a band.

According to our positioning, the IMU provides, respectively, ulnar and radial deviation (yaw), flexion and extension (pitch), and pronation and supination of forearm. The subjects were instructed to sit up straight, feet flat on the floor, with the examined arm fixed using an elastic band. The IMU has a wireless connection with a laptop where specific software can provide in real time the 3D kinematics of wrist.

**FIG. 1.**
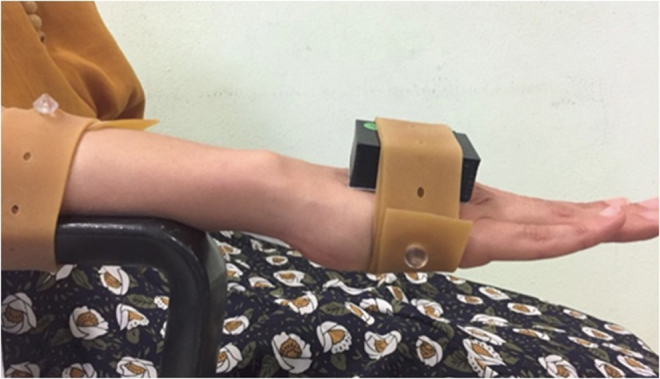
Position of the IMU. IMU, inertial measurement unit.

After an anatomical calibration and a brief warm-up period, each subject performed flexion (FL)-extension (EX), radial (RD), and ulnar deviation (UD). After these movements, the IMU was positioned on the dorsal surface of the radio with the forearm in a neutral position, and pronation (PR) and supination (SU) were performed (Fig. 1).

Movements on each plane were repeated four times to obtain an average value for each measurement. For each trial, the maximal values of FL, EX, RD, UD, PR, and SU were calculated. We evaluated both the affected and unaffected wrist. We calculated the value of ROM as a percentage with respect to the unaffected wrist of each patient and the ROM of the affected side to have information about the ROM within which percentage is calculated. With the IMU, it is possible to evaluate simultaneous movements on the three planes of space. This is why we calculated the value of RD and UD at the peak intervals (RDP) (UDP) of movements during FL and EX of the wrist as a CM.

**FIG. 2.**
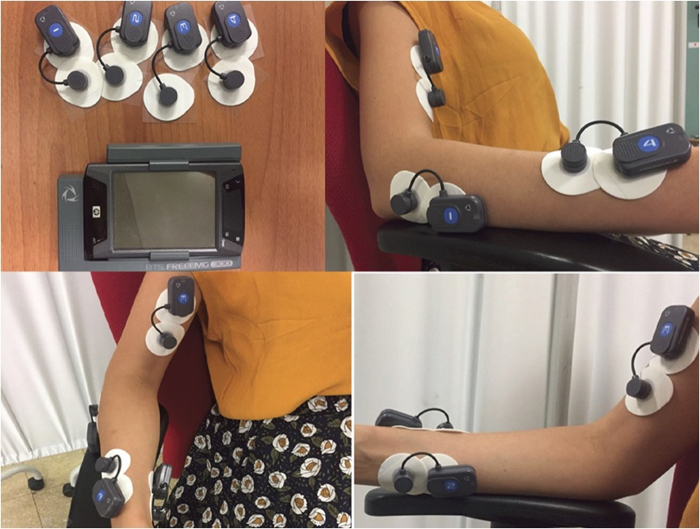
Surface EMG. EMG, electromyography.

We determined the presence of ulnar or radial compensation if there was an RD or UD value higher than 5°, respectively.^6^ If not, we determined it to be neutral deviation peak. We also recorded surface EMG signals using a multichannel Pocket Free EMG system (BTS) operating at a sampling rate of 1000 Hz, and band-pass filtered at 10–500 Hz (Fig. 2). EMG activity was recorded using pairs of Ag-AgCl surface electrodes precoated with electro conductive gel (diameter 1 cm, distance between electrodes ≤2 cm).

After careful shaving and cleansing of the skin with alcohol, electrodes were placed, in accordance with the recommendations from the SENIAM project,^10^ over biceps brachialis, flexor carpi radialis (FCR), extensor carpi radialis (ECR), and pronator teres (PT) muscles on the basis of standard anatomical landmarks.^11^ The subject was placed in the same position used for IMU acquisition, with his/her arm in adduction position, elbow flexed at 90°, and with his/her forearm in a neutral position (Fig. 2). Then, for each muscle, the subjects performed four isometric contractions, each lasting 30 sec, and maximal voluntary contractions.

We did a time-frequency transformation using Discrete Fourier Transform to analyze shifts in instantaneous SMF between each isometric contraction. The frequency spectrum of the EMG signal is a very sensitive indicator of MF; moreover, it has been demonstrated that a shift in the power spectrum of the surface EMG signal toward lower frequencies takes place as the muscle becomes fatigued.^12^ The isometric contraction was subdivided into intervals that last 1 sec, where the SMF were calculated. The percentage decrease in the median frequency is correlated to MF. The HGS was measured using a hand-held Jamar Hydraulic Hand Dynamometer (BASELINE Medical NY, USA).

The subject held the dynamometer while seated, with his/her shoulder adducted, forearm rotated neutrally, and elbow flexed at 90° and with the wrist in FL position. We tested both the healthy and the affected side three times; then we considered the average value for each side, and we calculated the value of HGS as a percentage with respect to unaffected wrist. The DASH questionnaire (range: 0–100 with 0 as the best result) and PRWE questionnaire were administered to the patients at the final follow-up.

The PRWE is a 15-item patient-reported questionnaire. It has two subscales: (1) Pain subscale: 5 items (responses ranging from 0 = no pain to 10 = worst ever) and (2) Function subscale: 10 items, which is further divided into: (a) Specific activities: 6 items (responses ranging from 0 = no difficulty to 10 = unable to do) and (b) Usual activities: 4 items.^13^ This study was developed in accordance with the STROBE guidelines.^14^

### Exclusion criteria

For the purpose of the study, we decided to apply the following exclusion criteria: (i) patients older than 75 and younger than 18 years of age, (ii) history of wrist fractures (involving articulation), (iii) bilateral distal radius fractures, (iv) fractures associated with nerve, vessels and tendon injury, (v) AO/ASIF B2, B3, and C3 fractures (Arbeitsgemeinschaft für Osteosynthesefragen/Association for the Study of Internal Fixation),^15^ (vi) radiographic evidence of preexisting wrist arthritis, (vii) rheumatoid arthritis, (viii) associated ulnar fracture, (ix) skeletal immaturity, (x) open fractures, (xi) patients with polytrauma or multiple upper limb injuries/fractures, and (xii) history of dementia or other cognitive and psychiatric disorders.

### Sample size

For the calculation of sample size, we used GP Power V.3.1.9.2; assuming the effect size of 1039 (5), *α* values of 0.05, power (1 − *β*) of 0.80, and an allocation ratio *N*2/*N*1 of 1; the sample size requested is 13 patients for each group.

### Statistical analysis

Statistical analysis was performed using SPSS IBM statistics v.24. The results are presented in terms of median and interval (minimum and maximum values). For all variables the normality of data were ascertained using the Kolmogorov-Smirnov Test. Since all parameters were not normally distributed, except fatigue, the Mann–Whitney *U* test was conducted to compare the kinematic values of the pulse on the three planes. The Mann–Whitney *U* test was conducted to compare the ROM of the affected wrist within the groups. *A chi square test* for independence was conducted between VPF and KWF and the type of CM during the flexion and extension movements. Significance was set at *p* = 0.05.

The Mann–Whitney *U* test was conducted to compare EMG data. The Mann–Whitney *U* test was used to evaluate DASH and PRWE for the two different groups. An independent sample *t*-test was run to determine if there were differences in HGS between the two groups. Significance was set at *p* = 0.05.

## Results

Between February 2013 and May 2017, 85 patients were treated in our hospital for distal radius fractures with KWF or VPF. After applying the exclusion criteria, 40 patients were recruited for our study and reviewed retrospectively. These patients had been treated for distal radius fractures (both intra-articular and extra-articular) with KWF (group A) or VPF (group B) at least 12 months before evaluation. Each patient was retrospectively classified on the basis of preoperative X-rays in accordance with the AO/ASIF system.^15^ In group A, 20 patients [9 males, 11 females, median age 55 (18–74); follow-up median 34 (13–69) months] had been treated with KWF after a closed reduction under c-arm guidance.

Patients were immobilized after surgery in a below-elbow cast and Kirschner wires were removed after 6 weeks. There were three A2 types, four A3 types, six C1 types, and seven C2 types. In group B, 20 patients [13 males, 7 females, median age 50 (17–72); follow-up median 26 (12–34) months] had been treated with AO distal volar locking plates (Synthes), which was performed using the distal Henry approach under tourniquet control. The restoration of the pronator quadratus was successfully carried out. Patients were immobilized after surgery in below-elbow cast for 2 weeks. After suture removal, patients were immobilized in a splint and a gentle approach to restoring of ROM was introduced.

In this group, there were two A1 types, four A2 types, three A3 types, seven C1 types, and four C3 types. For both procedures, patients were instructed to follow a similar rehabilitation program consisting of active and passive exercises of the wrist for a minimum of 1 h a day for 3 months.

### Kinematic data

There were no statistically significant differences among two groups: flexion (*U* = 353; *Z* = −1.137; *p* = 0.255); in extension (*U* = 384; *Z* = −0.967; *p* = 0.90); in radial deviation (*U* = 379; *Z* = −0.884; *p* = 0.377); and in pronation (*U* = 536; *Z* = 1.391; *p* = 0.164).

There were significant differences between the two groups: UD was significantly higher for VPF (mean rank = 36.25) than for KWF (mean rank = 24.25) (*U* = 605; *Z* = 3.392; *p* = 0.017); supination was significantly higher for VPF (mean rank 35.89) than for KWF (mean rank = 25.05) (*U* = 589; *Z* = 2.158; *p* = 0.031). In Table 1, we have reported the values of kinematic data expressed as a percentage with respect to the unaffected wrist.

**Table 1.** Range of Motion Expressed as a Percentage with Respect to the Unaffected Wrist

ROM	KWF	VPF	*P*
FL	90.42 (68.43–97.54)	96.26 (64.72–101.93)	0.255
EX	73.26 (45.17–96.67)	90.07 (63.85–101.83)	0.90
RD	85 (82.68–98.77)	92.29 (42.44–100)	0.377
UD	71.80 (67.93–98.53)	89.44 (60.23–100.62)	0.017^*^
PR	95.50 (55.44–99.91)	93.46 (79.98–100)	0.164
SU	82.87 (66.94–99.52)	89.64 (66.82–101.75)	0.031^*^

^*^ Significative.

EX, extension; FL, flexion; KWF, Kirschner wire fixation; PR, pronation; RD, radial deviation; SU, supination; ROM, range of motion; UD, ulnar deviation; VPF, volar plate fixation.

In Table 2, we have reported differences between the ROM of the affected wrist within the groups. There were no significant differences between the two groups: in flexion (*U* = 499; *Z* = 0.855; *p* = 0.393); in extension (*U* = 449; *Z* = −0.183; *p* = 0.90); in UD (*U* = 448; *Z* = 0.116; *p* = 0.908); and in supination (*U* = 560; *Z* = 1.738; *p* = 0.082).

**Table 2.** Range of Motion of Affected Wrist

ROM	KWF	VPF	*p*
FL	62.97 (36.44–77)	60.63 (50.57–82.57)	0.393
EX	97.523 (45.17–112.39)	96.17 (64.72–113.61)	0.90
RD	28.19 (18.19–37.63)	21.37 (18–39.19)	<0.001^*^
UD	28.68 (25.80–38.37)	30.91 (18–39.19)	0.908
PR	66.44 (50.63–81.07)	76.93 (63.36–92.06)	<0.001^*^
SU	64.18 (41.93–92.12)	76.66 (54.06–90.75)	0.82

^*^ Significative.

There were significant differences between the two groups: radial deviation was significantly higher for KWF (mean rank = 45.25) than for VPF (mean rank = 26.75) (*U* = 185; *Z* = −3.694; *p* < 0.001); pronation was significantly higher for VPF (mean rank = 35.89) than for KWF (mean rank = 25.05) (*U* = 707; *Z* = 3.868; *p* < 0.001).

A *chi square test* for independence was conducted between VPF and KWF and the type of CM during the flexion movement. There was no statistically significant association between type of surgical intervention and the type of CM, *χ*^2^ = 0.97; *p* = 0.953.

A *chi square test* for independence was conducted between VPF and KWF and the type of CM during the extension movement. There was no statistically significant association between type of surgical intervention and the type of CM, *χ*^2^ = 0.042; *p* = 0.979. We reported the value for each group expressed as a percentage of UDP and RDP during flexion and extension.

**FIG. 3.**
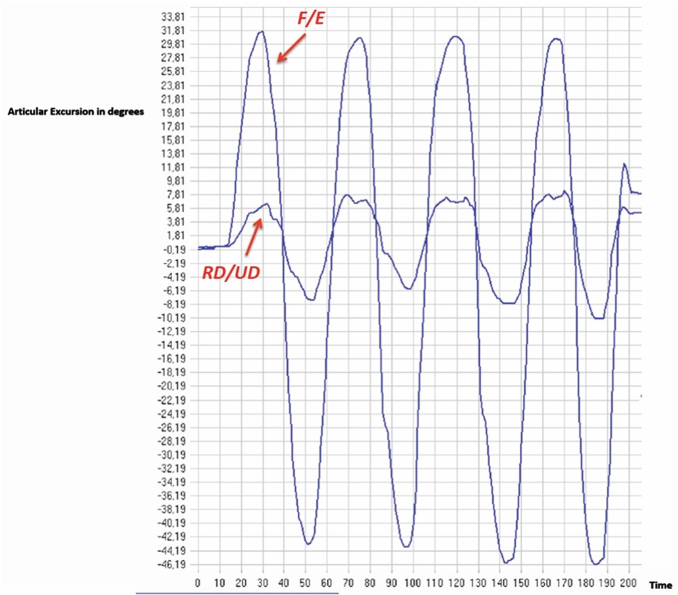
Example of CM during FL/EX. CM, compensatory movement; EX, extension; FL, flexion.

In Figure 3, we show an example of CM during FL/EX.

We found no deviation in 20% and 19% of patients, in KWF and VPF, respectively, during extension peak (Table 3) and no deviation in 20% and 20% of patients, in KWF and VPF, respectively, during flexion peak (Table 4).

**Table 3.** Extension Compensation Expressed as Percentage Neutral Deviation Peak, Radial Deviation Peak, Ulnar Deviation Peak

ROM	KWF (%)	VPF (%)
NDP	20	19
RDP	40	36
UDP	40	45

NDP, neutral deviation peak; RDP, radial deviation peak; UDP, ulnar deviation peak.

**Table 4.** Flexion Compensation Expressed as Percentage Neutral Deviation Peak, Radial Deviation Peak, Ulnar Deviation Peak

ROM	KWF (%)	VPF (%)
NDP	20	20
RDP	60	40
UDP	20	40

### EMG data

There was no statistically differences among the two group regarding: SMF of biceps brachialis (*U* = 45; *Z* = −0.507; *p* = 0.645); SMF ECR (*U* = 43.5; *Z* = −0.616; *p* = 0.547); SMF PT (*U* = 35.5; *Z* = −0.702; *p* = 0.492).

SMF FCR of volar plate were significantly higher (mean rank = 13.54) than KWF (mean rank = 6.88) (*U* = 85.5; *Z* = 2.390; *p* = 0.017). The results are expressed as a decreased percentage in the SMF.

**FIG. 4.**
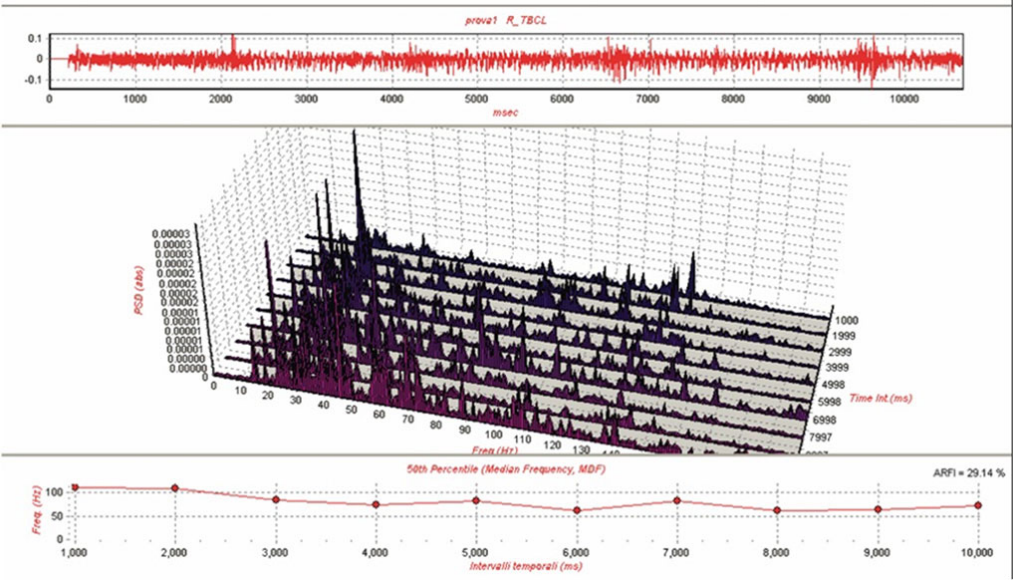
Example of electromyographic potentials during acquisitions.

In Figure 4, we have reported an example of electromyographic potentials during acquisitions (Table 5).

**Table 5.** Percentage Decrease in the Median Frequency Spectrum Median Frequency in: Biceps Brachialis, Extensor Carpi Radialis, Pronator Teres, and Flexor Carpi Radialis

SMF	KWF	VPF	P
BB	8.42 (0.69–16.77)	7.23 (−5.20–25.94)	0.645
ECR	9.69 (4.65–18.60)	7.22 (2.41–22.26)	0.547
PT	8.42 (−7.80–25.86)	5.50 (−7.80–16.21)	0.492
FCR	7.655 (−1.70–12.37)	15.62 (−5.92–44.46)	0.017^*^

^*^ Significative.

BB, biceps brachialis; ECR, extensor carpi radialis; FCR, flexor carpi radialis; PT, pronator teres; SMF, spectrum median frequency.

### Clinical evaluation

There were no significant differences between the two group as far as DASH (*U* = 38.5; *Z* = −0.980; *p* = 0.336) and PRWE (*U* = 34; *Z* = −1.320; *p* = 0.210). The HGS of KWF (93.90 ± 14.800) was significantly higher than VPF (78.569 ± 14.44) *p* = 0.037.

In Table 6, we have reported the values of DASH and PRWE and the value of HGS expressed as a percentage with respect to the unaffected wrist.

**Table 6.** The Disabilities of the Arm, Shoulder, and Hand Questionnaire Patient-Rated Wrist Evaluation Hand Grip Strength Expressed as a Percentage

Clinical evaluation	KWF	VPF	*P*
DASH	15 (0–37.15)	100 (69.7–112)	0.336
PRWE	19.5 (0–26)	4.3 (0–33)	0.210
HGS	93.90 ± 14.80	78.569 ± 14.44	0.037^*^

^*^ Significative.

DASH, disabilities of the arm, shoulder, and hand questionnaire; HGS, hand grip strength; PRWE, patient-rated wrist evaluation.

## Discussion

The aim of our study was to evaluate ROM recovery of the wrist, with the use of IMU, and to evaluate the onset of muscle fatigue during movements of the wrist and forearm, with the use of the surface-EMG, after at least of 1 year from surgical stabilization of distal radius fracture performed with VPF or KWF. In fact, currently surgical stabilization with KWF is the intervention most used to treat displaced distal radius fracture; however, the introduction of VPF is changing the contemporary approach.^16–19^

We performed a dynamic and 3D evaluation of the differences between the two groups of the patients with the IMU. This device is generally made up of a three-axis accelerometer that measures linear acceleration, a three-axis gyroscope measuring angular acceleration, and a three-axis magnetometer that determines the magnetic north to compensate for the orientation drift, which when combined together allow for an extremely accurate dynamic orientation.

The use of inertial motion sensors is an effective method to measure the joint range in an outpatient setting and such invention was a technological advancement in the field of biomechanics and wearable sensors. Unlike the use of optical detection systems in evaluating body movements, inertial systems do not require an external physical reference system.^4,20^

To our knowledge, there are no studies to date that evaluate wrist ROM using wearable devices such as IMU and this is the first study that examines wrist tridimensional ROM differences in patients with former radial distal fracture treated with VPF and KWF. In our study, we calculated the ranges of the affected side to give an idea of the ROM in which we worked. We found statistically significant differences between the two groups in two parameter: higher in RD for KWF compared to the VPF and in PR higher in VPF respect to KWF, probably due to an individual differences of starting condition at the time of trauma and due to the variability of the joint excursion of the population, as expressed in literature.^1,21^

Goehre et al.^19^ reported an after-surgery RD and UD data at 6 months, so they were not able to perform a pooled analysis on RD and UD at that time. There was a significant difference in SU favoring VPF at 3 and 6 months, but not at ≥12 months postoperatively. These data partially confirm our results, but ROM data of these studies were not obtained by IMU.

We measured ROM of the affected side, expressed as a percentage with respect to the healthy contralateral, to avoid potential bias due to individual variability. Our results do not show differences between the two groups, as far as FL and EX, RD and PR movements, as described in the literature, the data were comparable^22^; however, there are statistically significant differences as far as SU and UD, which are higher in the VPF group than the KWF group. Our study included patients with a longer follow-up and, in addition, our data showed significant differences in terms of UD as well. This aspect is probably due to the greater precision of the IMU technology that could better identify small differences than a standard goniometer evaluation.^23^

Costa et al.^7^ evaluated 461 patients having surgery for an acute dorsally displaced fracture of the distal radius, to either percutaneous KWF or VPF, and they found no statistically significant differences in the PRWE values at 3, 6, and 12 months after surgery.

Longxiang et al.^22^ also, showed that there are no significant differences between VPF and KWF in the treatment of distal radius fractures at 3, 6, and 12 months, after surgery (all *p* > 0.05). Our results are supported by the literature, where we found no statistical differences between the two groups as far as DASH and PRWE at >12-month follow-up. Although this follow-up is not long enough for subjects to develop posttraumatic arthritis, studies with greater long-term follow-up are necessary.

Furthermore, Rozental et al.^20^ evaluated 45 patients treated with VPF or KWF. Results demonstrated better results for the VPF immediately after surgery in terms of ROM and DASH score improvement. However, at the 12 months follow-up results were similar for the two groups. Therefore, the open approach could be more effective in terms of bone realignment, leading to better clinical outcomes and lower incidence of posttraumatic osteoarthritis, but at the 1-year follow-up after surgery, this does not appear to affect functional outcome.

IMU allows for the simultaneous evaluation of the association between various types of movements. We evaluated the presence of RD and UD during FL and EX movements to investigate the possible presence of CM in the two groups. We did not find a statistically significant correlation of the movements between the two groups.

Furthermore, to have more information about the functional status, we also investigated HGS using a dynamometer. Longxiang et al.,^22^ in their meta-analysis concerning HGS, demonstrated that VPF was superior to KWF at 3 and 6 months postoperatively. However, at ≥12 months, HGS was similar for the two treatment methods. Our results showed that HGS is statistically higher in patients who underwent KWF as opposed to VPF, during FL contraction of the wrist. Perhaps, these results are related to the surgical technique.

In fact, Duque et al.^24^ showed that HGS, in flexion, required a much greater engagement of muscle flexors than HGS in extension. This could explain our results, meaning the possible muscle damage during volar open access could reduce in VPF group the HGS. During the examinations, patients underwent also an EMGs evaluation, to detect muscle activation and MF. Our results showed a higher incidence of MF, during isometric flexion contraction, in the VPF group than the KWF group.^8^

This is probably due to the bigger trauma that is an intrinsic characteristic of the open surgery technique, leading to a higher energy expenditure during endurance tasks. Furthermore, after a wrist fracture, it could be difficult return to work, especially if the patient engages in an activity that could lead to repetitive joint overload. Although, there is no available data about EMG evaluation in patients who underwent a surgical intervention for wrist fracture. Some studies have demonstrated a direct relationship between muscle activity amplitude and pain.^25^

Aslani et al.^26^ reported the study of shoulder ROM with a single IMU combined with an EMG sensor to monitor the 3D reachable workspace with simultaneous measurement of deltoid muscle activity, showing that shoulder disorders may be assessed in terms of 3D surface area and with EMG.

In the past decade, several studies have been done to analyze the field of application for wearable sensors. The wearable IMU may also be used to quantify and monitor progress of a rehabilitation program.

Wang et al.,^27^ in their systematic review, underline the role of IMU to value the ROM assessment and body segment position, during upper body rehabilitation. The interpretation of these values is crucial for the development of therapy treatment by the clinicians.

O'Reilly et al.,^28^ in their recent review, demonstrated the strong reliability of the use of wearable IMU for measurement of joint angle and ROM during lower limb exercise, also comparing these results with the values of the standard measurements.

van Meulen et al.,^29^ in a pilot study, proposed an algorithm with the use of single IMU attached to the forearm, which could be used during clinical evaluation or functional tasks.

Chen et al.^30^ used sensors to evaluate short-arc exercise, straight leg raise, and quadriceps strengthening mini-squats in patients with knee arthritis with good experimental results, being able, 1 day, to help the patient in the right way to perform rehabilitation movements.

Chiang et al.^31^ applied the wearable IMU for valuation of ROM of knee after knee joint replacement, emphasizing the possibility, with technological progress, to evaluate articular recovery after surgical procedures, no longer with classical methods, susceptible to individual judgment, but with objective methods. Was also investigated the possibility of using the IMU sensor to limit errors during the positioning for hip replacement.^32^

The reproducibility and validity of measurements with IMU sensor have been demonstrated by comparing them with accepted technology. This gold standard has to be a tool for measuring human movement, providing an excellent option for motion analysis.^33^ With technological progress in many fields, the interest in technology applied to movement analysis is arousing interest. It has been shown that inertial sensors can be used for many different purposes, from motion analysis to assess joint recovery after surgery to the evaluation of rehabilitative activity, for identifying movement disorders or quantify and monitor progress of a rehabilitation program, also with the installation of application on the smart-phone.

With the IMU devices, in the future, it will probably be possible to assess movement disorders outside the constrained environments of hospitals and research laboratories. Patient progress could be easily recognized by the surgeon during rehabilitation. Also, the IMU and EMG evaluations could be easily performed in outpatient clinics, or also allow for long distance follow-ups using telemedicine systems.^34^

There are some limits to our study. The first limit is those related to the technical aspects of the inertial sensors. In fact, their accuracy can be influenced by factors such as humidity and temperature as well as the presence of metal objects or mobile phone can alter the transmission of the signal; also, they cannot be applied to patients with metallic implants. The second limit is inherent to the small sample size. The large standard deviations for the age and follow-up make comparison among subjects difficult. Moreover, using a single IMU to evaluate a wrist's ROM could lead to some difficulty in the registration that should be avoided using two IMUs.

However, we decided to use only one IMU since it was the easiest way to perform the examination, to make this technique more applicable in daily clinical practice and also for his miniature in size and lower power consumption.

## Conclusion

We focused on the comparison between open reduction and internal fixation with a volar locking plate and closed reduction with percutaneous KWF procedures, the two most common surgical options utilized for the treatment of distal radius fractures.

In literature, at 12 weeks from the procedure, clinical results seem to favor patients treated with plating, but there were no significant differences between the two types of treatment at long-term follow-up. Our results demonstrate a superior efficacy of VPF in terms of ROM improvement in UD and supination, but these patients' muscle fatigue is greater than the KWF group. However, these results are not related to clinical functional assessment tests.

Our results are comparable with the literature, where it is evident that there are no differences between two surgical procedures in terms of functional recovery, although the methods used are not overlapping. In fact, the differences founded in terms of ROM in UD and in supination may be due from IMU sensor utilization compared to other static evaluations. Moreover, the IMU sensor could be used, in the future, to evaluate ROM after surgery during patient's rehabilitation.

In conclusion, based on the data available, volar locking plate fixation is similar to percutaneous fixation for the treatment of distal radius fractures. We suggest further studies with the use of two IMU sensors to compare the effects of volar locking plate with percutaneous fixation in patients with stratified analysis regarding age and fracture type, paralleled with cost-effectiveness analysis.

## Abbreviations Used

BB: biceps brachialis
CM: compensatory movement
DASH: disability of the arm, shoulder, and hand
ECR: extensor carpi radialis
EMG: electromyography
EX: extension
FCR: flexor carpi radialis
FL: flexion
HGS: hand grip strength
IMU: inertial measurement unit
KWF: Kirschner wire fixation
MF: muscle fatigue
NDP: neutral deviation peak
PR: pronation
PRWE: patient-related wrist evaluation questionnaire
PT: pronator teres
RD: radial deviation
RDP: radial deviation peak
ROM: range of motion
SMF: spectrum median frequency
UD: ulnar deviation
UDP: ulnar deviation peak
VPF: volar plate fixation
